# Associations of Microbiota and Nutrition with Cognitive Impairment in Diseases

**DOI:** 10.3390/nu16203570

**Published:** 2024-10-21

**Authors:** Ewelina Młynarska, Paulina Jakubowska, Weronika Frąk, Agata Gajewska, Joanna Sornowska, Sylwia Skwira, Jakub Wasiak, Jacek Rysz, Beata Franczyk

**Affiliations:** 1 Department of Nephrocardiology, Medical University of Lodz, ul. Zeromskiego 113, 90-549 Lodz, Poland; 2 Department of Nephrology, Hypertension and Family Medicine, Medical University of Lodz, ul. Zeromskiego 113, 90-549 Lodz, Poland

**Keywords:** cognitive impairment, diet, microbiota

## Abstract

Background/Objectives: Recent research highlights the growing interest in the impact of nutrition on cognitive health and function in disease, as dietary habits are increasingly recognized as crucial factors in relation to brain function. This focus is especially important given the rising prevalence of neurodegenerative diseases and the cognitive decline associated with poor dietary choices. Links are now being sought between brain function and the microbiota and gut–brain axis. Mechanisms are proposed that include low-grade chronic neuroinflammation, the influence of short-chain fatty acids, or the disruption of glial cells and transmitters in the brain. Methods: We reviewed the articles on pubmed. This is not a systematic review, but of the narrative type. We wanted to outline the issue and summarise the latest information. Results: The axis in question has its foundation in nutrition. It has been reported that diet, particularly the components and the timing of food intake, has an impact on cognitive processes. The Mediterranean diet is most often cited in the literature as being beneficial to health. In order to obtain a more complete view, it is worth considering other dietary patterns, even those that impair our health. Conclusions: Determining what is beneficial and what is not will allow us to develop a speronized strategy for the prevention of, and fight against, cognitive impairment. Appropriately selected supplements, the functions of which we have also discussed, may prove supportive.

## 1. Introduction

The relationship between dietary intake and cognitive function has garnered increasing attention in recent years, particularly considering the increasing incidence of neurodegenerative and mental health disorders, both of which are closely linked to cognitive impairment. Cognitive functions encompass a wide range of mental processes essential for navigating daily life and maintaining overall well-being. These functions include memory, attention, language, executive function, and perception. In everyday life, various factors can contribute to cognitive function, influencing individuals’ well-being. These include sleep quality, physical activity, stress levels, engagement in cognitively stimulating activities, and social interactions. Additionally, dietary habits play a crucial role, as the nutrients we ingest can significantly affect our brain function, influencing aspects such as memory, attention, and overall cognitive health. This article aims to examine the existing literature on cognitive impairment associated with various diseases and the effects of different dietary patterns and nutrients on distinct cognitive functions in these disorders. Furthermore, it identifies potential avenues for future research in this domain.

## 2. Cognitive Dysfunctions and Their Underlying Mechanisms in Various Diseases

**Figure 1:**
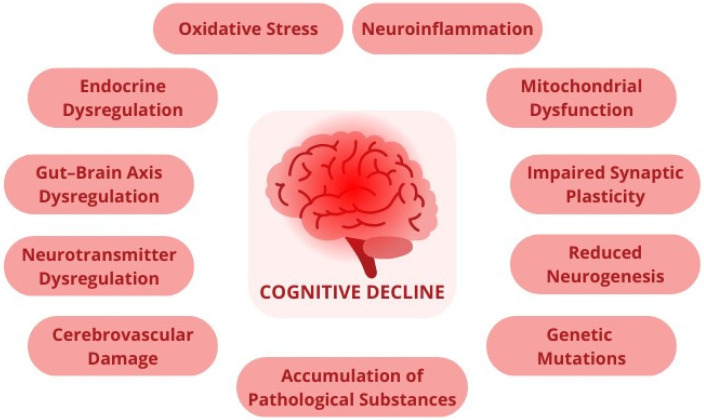
This figure summarizes the primary mechanisms contributing to cognitive dysfunction across various diseases, highlighting the complexity and interconnectivity of these processes.

Cognitive functions are essential for an individual’s capacity for managing daily life, which involves a variety of mental processes such as memory, attention, executive function, and problem-solving. However, cognitive impairment is a hallmark of several health conditions, including pathological aging, neurodegenerative diseases, psychiatric disorders, nutritional disorders and obesity. These conditions affect cognitive function by specific but often overlapping mechanisms, leading to various levels of impairment (Figure 1).

### 2.1. Cognitive Function and Aging

Cognitive decline is a natural part of aging, with most individuals experiencing some deterioration in memory, processing speed, and executive function, driven by several mechanisms. One of the main causes is a decrease in the hippocampus volume, which impairs memory storage and learning [1]. Additionally, oxidative stress, which naturally increases with aging, plays a crucial role in cognitive decline as the accumulation of reactive oxygen species (ROS) coupled with reduced antioxidant defenses causes oxidative damage to neurons. This process is further exacerbated by age-related mitochondrial dysfunction, leading to additional oxidative stress [2]. Furthermore, decreased neuroplasticity, accompanied by decreased synapse formation and neurogenesis in the hippocampus, limits the brain’s ability to adapt to new cognitive challenges, leading to progressive cognitive decline over time [3].

### 2.2. Accelerated Cognitive Decline in Pathological Aging

Pathological aging is characterized by several mechanisms that accelerate cognitive decline beyond what is typically observed in normal aging. One significant factor is enhanced neuroinflammation, where chronic activation of the microglia and astrocytes leads to persistent brain inflammation, which exacerbates neuronal damage and accelerates cognitive decline. Additionally, mitochondrial dysfunction increases reactive oxygen species (ROS) production, which heightens oxidative stress and further exacerbates neuroinflammation, creating a vicious cycle that accelerates neuronal damage and cognitive decline [4]. Additionally, cerebrovascular damage contributes to pathological aging by impairing the cerebral blood flow, which results in ischemic injury to neurons and further compromises brain function [5]. Another significant factor is the increased accumulation of pathological substances and impaired clearance mechanisms, which disrupt neuronal function and synaptic communication, thereby leading to severe cognitive impairments [6,7].

### 2.3. Cognitive Function in Neurodegenerative Disorders

The most common neurodegenerative diseases, including Parkinson’s disease (PD), Alzheimer’s disease (AD), frontotemporal dementia and multiple sclerosis, exhibit varied clinical profiles and underlying pathophysiology. However, they generally share a common feature: cognitive impairment, which progressively worsens as the disease advances. These impairments can manifest as difficulties with memory, concentration, spatial orientation, and the ability to perform complex tasks, as well as changes in behavior and personality. As the disease progresses, these issues usually lead to increasing challenges in everyday life.

In patients with Parkinson’s disease, neuropsychological assessments often reveal mild to moderate impairments in visuospatial skills, attention, and working memory. The evidence consistently suggests that low cerebrospinal fluid levels of amyloid-β42, a marker of comorbid Alzheimer’s disease, predict future cognitive decline and dementia in Parkinson’s disease [8].

The presence of two abnormal structures, senile plaques and neurofibrillary tangles, occurs before the neuronal death and brain degeneration seen later in Alzheimer’s disease. The plaques consist of beta-amyloid peptide aggregates that progressively form in a specific pattern as the disease develops. This pattern begins in the neocortex and spreads through the allocortex, hippocampus, basal ganglia, midbrain, and cerebellum. Neurofibrillary tangles, which are aggregates of the tau protein, also develop later and spread along a different pathway, starting in the transentorhinal region and extending through the entorhinal cortex, hippocampus, and neocortical areas. Thus, there are two distinct pathologies—amyloid and tau—each with different structural changes that appear at different stages of the disease. It has been suggested that the disorder may only be classified as a unique disease when both the amyloid and tau pathologies converge. Prior to this overlap, distinct features of cognitive dysfunction may be associated with the presence of plaques in specific brain regions. Memory loss, cognitive decline, impairment of executive functions, and loss of consciousness, among other symptoms, are observed in AD. These symptoms can emerge at various stages throughout the progression of the disease, gradually advancing along a continuum that ends in dementia and extensive brain degeneration.

### 2.4. Cognitive Function in Psychiatric Disorders

Cognitive dysfunction, which impacts attention, memory, executive function, and social cognition, is prevalent across various psychiatric disorders. While some mechanisms differ, there are common pathways that may underlie these cognitive impairments.

Disruptions in neurotransmitter systems, including dopamine, serotonin, and glutamate, are central to cognitive impairments in psychiatric disorders. Imbalances in dopamine transmission, such as hypoactivity in the prefrontal cortex and hyperactivity in the mesolimbic pathway, contribute to deficits in executive function and working memory, which are evident in disorders like schizophrenia [9,10]. Alterations in serotonin levels or receptor function can impair mood regulation and cognitive processes such as memory and attention, a common issue in major depressive disorder [11]. Additionally, alterations in glutamatergic and GABAergic neurotransmission impair cognitive processes in disorders like schizophrenia and mood disorders [12].

Another mechanism of cognitive impairment in psychiatric disorders may be neuroinflammation, which involves chronic activation of immune responses in the brain [13]. Elevated levels of pro-inflammatory cytokines, such as IL-6, TNF-alpha, and IL-1β, contribute to cognitive deficits by activating the microglia and astrocytes, disrupting synaptic plasticity, impairing neurogenesis, and compromising the integrity of the blood–brain barrier [14,15]. This inflammatory environment is often exacerbated by dysregulation of the hypothalamic–pituitary–adrenal (HPA) axis, which results in sustained high cortisol levels. Elevated cortisol further aggravates neuroinflammation, leading to increased neuronal damage and cognitive decline [13].

Increased oxidative stress, marked by an imbalance between the production of ROS and the body’s antioxidant defenses, plays a pivotal role in the neuronal damage and cognitive dysfunction observed in psychiatric disorders. Excessive ROS can lead to the oxidation of lipids, proteins, and DNA, particularly in critical brain regions essential for cognitive processing [16]. Furthermore, oxidative stress impairs mitochondrial function, leading to mitochondrial DNA (mtDNA) dysfunction, which further aggravates ROS production. This creates a vicious cycle where damaged mitochondria produce even more ROS, exacerbating the oxidative damage and contributing to the progressive decline in neuronal function and cognitive abilities [17]. Additionally, oxidative stress can exacerbate neuroinflammation by further activating the microglia and promoting the release of pro-inflammatory cytokines, creating a feedback loop that intensifies the neuronal damage and cognitive impairment [18].

### 2.5. Cognitive Function in Dietary Diseases

Dietary diseases, including type 2 diabetes, celiac disease and malnutrition, have been increasingly recognized for their significant impact on cognitive function. These conditions, often resulting from poor nutritional choices, disrupt normal metabolic processes, which can impair cognitive abilities.

In the case of type 2 diabetes, hyperglycemia, insulin resistance, and chronic inflammation are key mechanisms through which cognitive function is compromised. These metabolic disturbances lead to neuroinflammation, oxidative stress, and vascular damage, all of which contribute to cognitive decline and an increased risk of developing neurodegenerative diseases such as Alzheimer’s disease [19].

Celiac disease presents another example where the autoimmune response to gluten in individuals with celiac disease can lead to neuroinflammation and cognitive dysfunction, often termed “brain fog”, which refers to a range of symptoms, including cognitive slowing, difficulty concentrating and problems with the short- and long-term memory [20].

Moreover, malnutrition, particularly in older adults, is a significant risk factor for cognitive decline. Nutritional deficiencies, such as in B vitamins, vitamin D, and essential fatty acids, are associated with impaired cognitive function and an increased risk of dementia. Malnutrition increases the susceptibility of the aging brain to neurodegenerative processes, thereby accelerating cognitive decline [21].

### 2.6. Cognitive Function in Obesity

The rate of obesity is significantly increasing, and alongside this trend, there is a corresponding rise in the occurrence of metabolic disorders such as cardiovascular disease, systemic hypertension and type 2 diabetes. Excessive adiposity leads to insulin resistance, systemic inflammation, and altered brain structure and function, especially within the fronto-mesolimbic circuitry [22]. Neuroimaging studies have consistently demonstrated that obesity is linked to reduced gray matter volume and altered functional connectivity, which are predictive of cognitive impairment. Research has shown that obesity doubles the risk of developing Alzheimer’s disease, and being obese during midlife is a strong predictor of a higher likelihood of dementia in later years [23].

A cross-sectional longitudinal study involving more than 2000 middle-aged workers found a consistent linear relationship between BMI and cognitive function, as measured by the word-list learning test, which assesses verbal learning and memory, and the digit-symbol substitution test, which evaluates attention, response speed, and visuomotor coordination [24]. The results showed that obese individuals recalled fewer words on the word-list learning test and required more time to complete the DSST compared to individuals of normal weight.

## 3. Microbiota and Gut–Brain Axis

One research topic that has been gaining popularity in recent years is the gut microbiota, the gut–brain axis (GBA), and their impact on whole body function, including brain activity and cognitive function. The term microbiota is used to describe the microorganisms, including commensal bacteria, that inhabit the human gastrointestinal tract. It is estimated that a person’s gut microbiome contains over 100 trillion bacteria, with their numbers increasing from the stomach to the intestines and colon [25]. While the specific composition of the microbiota varies among individuals, healthy people typically share similar proportions: 60% to 80% are Firmicutes, 20% to 40% are *Bacteroidetes*, and around 5% consist of *Proteobacteria* and *Actinobacteria* [26,27]. Microbiota development starts in the fetal stage and is strongly influenced by the mode of delivery, whether vaginal or cesarean. Additionally, the composition of the microbiota is affected by the infant’s diet, particularly whether they are breastfed or formula-fed [28].

Acute changes in a person’s diet account for about 20% of the variability in the human microbiota, but long-term dietary habits have a more significant impact on the microbiota composition [29]. The types of food we consume influence the growth of specific bacterial species, leading to two main enterotypes of microbiota. The first enterotype, characterized by a high presence of *Bacteroides*, is typical of the Western diet in highly industrialized countries, where low-fiber and high-fat diets are common. Within this enterotype, a subgroup, sometimes called Enterotype 3, is distinguished by a high abundance of *Ruminococcus*. The second enterotype, dominated by *Prevotella*, is common in less industrialized countries, where diets include less processed food and more fiber [30,31].

**Figure 2:**
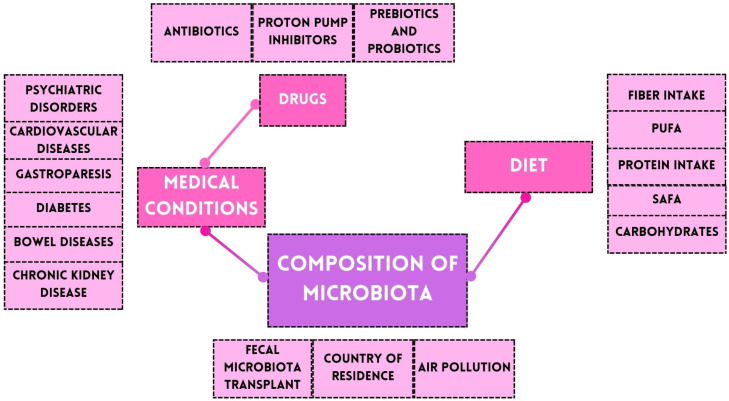
Factors affecting the microbiota composition.

It is worth noting that the composition of the microbiota is individual and is affected by many factors, such as the country of residence and environmental pollution. Any diseases and medical conditions present are also of great importance. A summary of the factors affecting the microbiota composition in adults is shown in Figure 2 [32,33,34,35,36,37,38,39,40].

The human gut microbiota and host share a symbiotic relationship, which implies that the host and microorganisms benefit from one another. The host provides a habitat and sustenance for microorganisms, which in turn benefit the host’s health by increasing disease resistance and nutrient absorption [41].

The GBA plays a crucial role in maintaining human health by enabling two-way communication between the gut microbiota and the brain. Achieving and maintaining eubiosis, which involves the natural presence of beneficial microorganisms, is vital for proper functioning. The GBA involves complex interactions between various systems, including the gut-associated immune system, the enteric nervous system (ENS), the vagus nerve, and the gut microbiota itself. These interactions are facilitated by the gut microbiota, which produces important compounds such as neurotransmitters, tryptophan, and short-chain fatty acids (SCFAs) that influence both gut and brain function [42,43,44].

Both the enteric nervous system (ENS), which is composed of glial cells and enteric neurons, and the autonomic nervous system, which is controlled by the vagus nerve, are involved in regulating immunological responses, motility, and gastrointestinal secretion. The body can independently affect the gut microbiota’s makeup thanks to these processes. In turn, gut microorganisms interact with the central nervous system (CNS) via immunological, hormonal, and neurological pathways, affecting the body [45,46,47]. For instance, gut bacteria can create short-chain fatty acids (SCFAs) from fiber, such as acetate and propionate, which can control the gene expression by hyperacetylating histones. The systemic effects of SCFAs include immune system modulation, appetite regulation, improved absorption of calcium, and preservation of glucose homeostasis [48,49]. SCFAs also have neuroprotective properties. Therefore, their depletion may lead to neurodegeneration [50].

Additionally, the microbiota influences CNS function by producing neurotransmitters (like acetylcholine, catecholamines, and gamma-aminobutyric acid) and biogenic amines (like histamine), as well as altering tryptophan metabolism [51,52,53].

The proper functioning of the gut–brain axis (GBA) relies on the integrity of the intestinal barrier, which separates the contents of the gut from the rest of the body, thus controlling the exchange of fluids and solutes. When dysbiosis occurs, this barrier is compromised, leading to increased intestinal permeability, often called “leaky gut” syndrome (LGS). This condition allows microbial components like lipopolysaccharide (LPS), toxic metabolites, and inflammatory substances to enter the bloodstream [54,55]. LPS is recognized by Toll-like receptors (TLRs), which trigger the release of pro-inflammatory cytokines such as tumor necrosis factor-alpha (TNF-α), IL-6, IL-8, and IL-12, causing both local and systemic inflammation [53,56,57]. According to current theories, the LPS in the CNS promotes amyloid-β and tau accumulation as well as neuropathology, which results in neurodegenerative illnesses [58]. Moreover, the administration of LPS activates the brain microglia, increases pro-inflammatory cytokines, and can cause depressive symptoms. Prolonged microglial activation results in complement-mediated loss of dopaminergic neurons and neurodegeneration [59,60].

Chronic systemic inflammation from LGS can disrupt the hypothalamus–pituitary–adrenal (HPA) axis, which is essential for supplying energy sources like glucose, amino acids, and free fatty acids to support immune function. This disruption leads to increased release of glucocorticoids and catecholamines, resulting in hypercortisolemia and overactivity of the HPA axis, which is linked to impaired glucocorticoid receptor function [33,61]. Additionally, increased intestinal permeability can activate T-cells, potentially leading to autoimmune disorders in the gut or other organs if these activated lymphocytes spread further [62].

Systemic inflammation disrupts the blood–brain barrier (BBB), impairing its ability to regulate the movement of substances, ions, and cells into the brain. This increased permeability of the BBB has been observed in several psychiatric disorders, including those associated with cognitive decline, such as Alzheimer’s disease [63,64]. BBB impairment and inflammation result in the decreased number and reduced function of astrocytes, a type of glial cell [65]. Astrocytes possess TLR4 receptors on their surface, which, upon detecting LPS, trigger an amplified proinflammatory response [66]. Additionally, astrocytes play a crucial role in regulating gamma-aminobutyric acid (GABA) and glutamate levels by participating in the reabsorption of these neurotransmitters [67].

### Influence of Gut Microbiota Metabolites and Neurotransmitters

Emerging evidence suggests that gut microbiota metabolites play a crucial role in the pathophysiology of neurodegenerative diseases such as Parkinson’s and Alzheimer’s.

LPS has been implicated in the exacerbation of neuroinflammation, suggesting its potential role in the pathogenesis of AD. The evidence supporting this hypothesis includes the elevated blood and brain LPS levels in Alzheimer’s patients, with LPS linked to increased amyloid-beta expression, aggregation, inflammation, neurotoxicity, tau phosphorylation, and microglial activation. Additionally, LPS may induce synapse and neuron loss, leading to memory deficits in AD animal models and cognitive dysfunction in humans [68].

In Parkinson’s disease, LPS has been shown to induce specific loss of midbrain dopaminergic neurons through microglial activation. Additionally, LPS induces α-synuclein expression, aggregation, and neurotoxicity, activates peripheral monocytes leading to inflammatory cytokine production, and mediates brain inflammation [69].

SCFAs, primarily produced by the gut microbiota, have garnered attention for their potential role in AD. SCFAs, particularly butyrate, acetate, and propionate, exhibit neuroprotective properties by enhancing neurotrophic factors like brain-derived neurotrophic factor (BDNF), which support neuronal health and synaptic plasticity. They also modulate neuroinflammation by inhibiting microglial activation and reducing pro-inflammatory cytokines, counteracting the inflammation associated with Alzheimer’s disease. Moreover, SCFAs help maintain blood–brain barrier (BBB) integrity, potentially preventing neurotoxic substances from entering the brain. Emerging evidence suggests that SCFAs may also influence amyloid-beta (Aβ) aggregation and tau phosphorylation, critical hallmarks of AD. Butyrate has been demonstrated to diminish the formation of neurotoxic amyloid-beta (Aβ) aggregates by inhibiting the assembly of the Aβ1–40 and Aβ1–42 peptides. In an animal model using 5xFAD mice, administering sodium butyrate (NaB) for twelve weeks led to a 40% decrease in the brain Aβ levels [70].

Some studies suggest that SCFAs may influence the aggregation of α-synuclein, a protein implicated in Parkinson’s disease. Additionally, SCFAs modulate neuroinflammation by inhibiting microglial activation and reducing pro-inflammatory cytokines, thereby protecting dopaminergic neurons from damage [71].

As mentioned, the microbiota also affects the production and metabolism of neurotransmitters. Firstly, recent research reveals that several neurotransmitters, including glutamate, GABA, serotonin, and dopamine, are among the metabolites generated by the gut bacteria [72]. GABA, an inhibitory neurotransmitter, was shown to be produced by certain bacteria species in the gut, such as *Parabacteroides, Eubacterium*, and *Bifidobacterium*, and act locally in the intestine [73,74]. The gut bacteria also create adenosine, which has local anti-inflammatory properties [75] and norepinephrine, which slows down the gut’s overall transit and reduces the number of migratory motor complexes. Additionally, it affects behavior and cognition, including learning, memory, and attention, and has an immunomodulatory impact [52,76].

Certain enzymes that can catalyze the conversion of substrates into matching neurotransmitters or precursors are encoded by genes found in certain bacteria. For example, SCFAs impact brain neurochemistry by controlling the expression levels of two enzymes: tyrosine hydroxylase, which is engaged in a rate-limiting step in the biosynthesis of dopamine, noradrenaline, and adrenaline, and tryptophan 5-hydroxylase 1, which is involved in the production of serotonin [77,78].

Moreover, the microbiota influences the metabolism of tryptophan, an essential amino acid, a precursor to serotonin (5-HT), which plays a vital role in central nervous system function. Imbalances in serotonin levels are closely linked to the development of mood disorders such as depression and anxiety. However, under inflammatory conditions, much of the tryptophan is diverted to the kynurenine pathway. LPS and pro-inflammatory cytokines activate enzymes like indoleamine 2,3-dioxygenase (IDO) and tryptophan 2,3-dioxygenase (TDO), leading to a reduction in serotonin production. Instead, tryptophan is metabolized into kynurenine, which can be further broken down into compounds like quinolinic acid (QUIN) and kynurenic acid (KYNA). While KYNA typically has neuroprotective functions, imbalances caused by inflammation can lead to neurodegeneration and cognitive issues. QUIN, an NMDA receptor agonist, contributes to excessive synaptic stimulation, causing neuronal damage [65,79,80].

## 4. Diet

One of the modifiable contributors that influence cognitive health is diet [81]. The correlation between eating habits and intellectual capacity continues to emerge in recent research. There is evidence showing that a variety of mental abilities, including memory, attention or decision-making, are influenced by the type of food consumed [82]. A decline in all these functions lead to conditions such as dementia or Alzheimer’s disease [83]. Acknowledging the relationship between these components is crucial for preventing a cognitive decline in patients by incorporating dietary patterns such as the Mediterranean or Mediterranean-DASH Intervention for Neurodegenerative Delay (MIND) diet in their daily life [81].

### 4.1. Mediterranean Diet

The Mediterranean diet (MD) is characterized by high consumption of antioxidants from fruits and vegetables and monounsaturated fatty acids (MUFAs) from olive oil or nuts [84]. It also consists of a moderate intake of polyunsaturated fatty acids (PUFAs) from fish and poultry, and low consumption of red and processed meat [84,85]. Components such as MUFAs and PUFAs have recently been highlighted in research for their positive effects on cognitive performance and decreased risk of the decline associated with aging [84]. Some studies have also indicated that consuming fruits and vegetables, which are one the main elements of the MD, is beneficial to mental abilities [83]. There are many mechanisms linking the MD to cognitive function. One of them is the anti-oxidative effect resulting from components of the MD diet such as olive oil and nuts that are high in phenolic compounds, which may help reduce oxidative damage in the brain and potentially prevent neurodegeneration [86]. Moreover, a reduction in the cardiovascular disease risk described in 1975 by Ancel Keys is strongly linked to cognitive function via a high intake of healthy fats, which improve heart function and ensure better overall blood flow to the brain [81,84,87]. The other effect of the MD on mental abilities might be enabled by the anti-inflammatory factor in the brain [84]. As reported by Gu et al., individuals who adhered most closely to the MD had substantially lower levels of high-sensitivity CRP [88]. The influence of the MD on cognitive health has also been [84] proved by Feart et al. in their study, where participants who followed the MD had a notably reduced number of errors on the Mini-Mental State Examination (MMSE) and higher scores in higher Free and Cued Selective Reminding Test (FCSRT) [85,89]. A few years later, a group of individuals from the same study went through 3-T magnetic resonance imaging that revealed importantly preserved white matter and an increase in structural connectivity in patients on the MD [90]. All of the benefits coming from implementing the MD are undisputed, as even the World Health Organization (WHO) has put the MD in its guidelines for reducing cognitive decline and the dementia risk [91].

What we eat affects our microbiome, which influences the brain. The Mediterranean diet has a multi-directional effect on the above-mentioned axis, e.g., by modifying the microbiome, which is more diverse than those following a Western diet. Additionally, switching to a Mediterranean diet increases the amount of Prevotella and Firmicutes bacteria, which correlates with an elevation of SCFAs and decreased inflammation [26]. In one of the more interesting studies, a diet rich in whole grains (WGs) markedly reduced TNF-α and LBP, while eating fruits and vegetables (FVs) led to a decrease in IL-6 and LBP [92].

### 4.2. The Mediterranean-DASH Intervention for Neurodegenerative Delay (MIND) Diet

The MIND diet has been developed by researchers at Rush University Medical Center and merges aspects of the Mediterranean and Dietary Approaches to Stop Hypertension (DASH) diets, aiming to preserve cognitive function and protect brain health through the aging process [87]. Developed by researchers at Rush University Medical Center, the MIND diet’s main aim is to promote brain health and reduce the risk of cognitive decline. As it is a combination of diets designed for managing hypertension, the MIND diet also has a positive influence on vascular health [81]. Moreover, it has attracted considerable interest due to its potential to slow the development of neurodegenerative diseases, such as a 53% lower risk of developing Alzheimer’s disease [87]. The main components of the MIND diet are green leafy vegetables, berries, extra-virgin oil, nuts, whole grains and lean protein sources [93]. Animal products and saturated fats are limited due to the association with inflammation and cognitive decline [94]. However, it uniquely focuses on eating berries and leafy greens, while not requiring a high fruit intake or more than one weekly fish meal, as in the Mediterranean and DASH diets [94]. The unique elements such as berries in studies have shown a protective influence on cognitive loss [95]. As research continues to explore this issue, the MIND diet stands out as a powerful tool for maintaining mental acuity and protecting against cognitive decline.

### 4.3. Vegetarian Diet

The relationship between diet and cognitive function has garnered increasing attention in recent years, particularly as researchers explore how various dietary patterns influence brain health. One of the areas of interest is the vegetarian diet. However, the evidence regarding the cognitive and mental effects of a plant-based diet remains uncertain [96]. A well-planned vegetarian diet is typically rich in nutrient-dense foods such as fruits, vegetables, whole grains, nuts, and seeds. It is typically low in saturated fats and cholesterol, promoting a healthier blood lipid profile. Moreover, it consists of foods high in polyphenols like cocoa, berries or nuts, which provide significant benefits for memory and cognitive functions, particularly in areas like attention and processing speed [97]. On the contrary, a plant-based diet may be deficient in certain vitamins, such as B12 and micronutrients, potentially leading to nutritional deficiencies. B12, also known as cobalamin, is crucial for maintaining neurological health and cognitive function. It is essential for the synthesis of myelin and is also involved in energy metabolism. Deficiency can impair cognitive functions, leading to issues like fatigue, poor concentration, and memory problems leading to dementia. Although a vegetarian diet brings in many nutritional components that have a positive impact on cognitive function, is it important to supplement any deficient nutrients during it, as this can provide significant benefits for the memory [98].

### 4.4. The Ketogenic Diet

The ketogenic diet is the intake of high-fat and low-carbohydrate foods. It leads to the formation and consumption of ketones as an alternative energy source. Interestingly, in Alzheimer’s disease, there are a reduced number of receptors in the brain for insulin, and there is also the phenomenon of brain insulin resistance [99]. There are studies, using scales such as the MMSE, ADAS-Cog and NM, supporting the thesis that a ketogenic diet can improve the cognitive function of people with Alzheimer’s disease. Undoubtedly, the disadvantage is the emerging hyperlipidemia, threatening cardiovascular health, and vitamin deficiencies [100].

One literature review assessed that for patients with Alzheimer’s, Parkinson’s and mild cognitive impairment, the ketogenic diet minimally improves cognitive function compared to the Mediterranean diet, modified Atkins diet and low-carbohydrate diet, or ketogenic medium-chain triglyceride (kMCT) supplementation [101]. It is currently considered preferable to use MCT oil and BOHB-containing supplements in addition to the standard diet than to use a ketogenic diet in patients with Alzheimer’s disease and Parkinson’s disease [102].

### 4.5. Time Restriction in Eating (TRE) and Intermittent Fasting (IFA)

TRE and IFA are dietary approaches aimed at reducing food intake throughout the day. Both lead to increased glucose uptake in the brain, which works to prevent neurodegenerative diseases, and also modulate the microbiome. IFA has been proven to have beneficial effects on synaptic plasticity, neurogenesis and BDNF-mediated neuroprotection [103].

In Alzheimer’s disease, this diet causes a decrease in β-amyloid, better synaptic adaptation in the hippocampus, and improved cognitive function, and it keeps the vascular system healthy [104].

Interestingly, intermittent fasting increases *Lachnospiraceae* and thus butyric acid, which has health-promoting effects [105].

### 4.6. Ultra-Processed Foods (UPFs)

Ultra-processed foods (UPFs) are favored by the public because of their low cost, time-saving nature and taste. The known and visible effects are obesity and cardiovascular disease [106]. However, what we take into our bodies also has consequences at a mental level. UPF includes, for example, salt, sugar, fats, hydrolyzed proteins, modified starches and hydrogenated or interesterified oil. When consumed in higher amounts, they induce low-grade systemic inflammatory and oxidative changes [107,108]. These changes are seen in the form of dysbiosis, which in turn affects the gut–brain axis [108]. Links have been established between modified foods and brain inflammation, anxiety–depressive behavior and cognitive decline [109]. In a cross-sectional study of a sample of 3632 participants over 60 years of age, it was found that in the animal fluency cognitive test to assess language and executive function in older people, the consumption of highly processed foods correlates with worse outcomes [110].

A recent meta-analysis of 28 studies found that long-term and excessive consumption of UPF has a negative impact on cognitive function, but it is not associated with an increased risk of Alzheimer’s disease or dementia [111]. A different conclusion was reached by the authors of a systematic review in which, after analyzing cohort studies, they found that UPF correlates with this neurodegenerative disease [112].

It has been shown in an animal model (mice) that long-term, 12-month exposure to advanced lipid peroxidation products (ALEs) contained in this food pattern significantly impairs cognitive functions related to the working memory, long-term memory, spatial memory and learning. The study also confirmed that the gut–brain microbiota remains an important link through interactions with ALEs [109].

Mitochondrial dysfunction is implicated in cognitive decline. The exact mechanisms are not fully understood, but it was shown that salt significantly inhibited mitochondrial division and blocked mitophagy, consequently disrupting mitochondrial function and negatively affecting synaptic plasticity. The protective factor was found to be SIRT3, which promotes mitochondrial division and promotes the PINK1/Parkin-dependent pathway associated with proper mitophagy [113].

There are reports that excess AGEs in the circulatory system and skin are linked to cognitive disabilities, dementia, as well as depression and schizophrenia. Underlying the pathophysiology is oxidative stress and neuroinflammation [114].

A study by Varun M. Bhave et al. showed that UPF negatively affects memory and fluency assessments in older adults, regardless of risk factors and adherence to recommended dietary patterns such as the MIND, MD, and DASH [115].

It is also essential to be aware that UPFs have different potential for harm among their categories, as shown by the NOVA classification. Based on the example of the Galit Weinstein study, highly processed meat and oils were shown to correlate with accelerated cognitive decline, such as executive functions and global cognition, in older people with type 2 diabetes, especially among women and obese people, while food calories did not find a link. In the same study, it was shown that UPF, originating from dairy products, bread, biscuits and starch, was not related to changes in cognitive function.

Intestinal dysbiosis may have its origins in the high-fructose diet that is part of the Western diet. Modification of the microbiota composition has been shown to be a critical factor in mouse hippocampal neuroinflammation. The conclusions from this study include a protective role for SCFAs against the defective NLRP6 inflammasome of the colon, which is responsible for impairment of the intestinal epithelial barrier [116].

It is considered that the use of this diet leads to damage to the intestinal barrier and thus the previously discussed increased intestinal permeability [117].

The negative effects include cognitive processes. One study evaluating the effects of a high-fat and high-sugar diet noted similarities such as increased Clostridiales compared to a normal diet. Increased Clostridiales and decreased Bacteroidales in high-fat diets were correlated with impaired cognitive flexibility. Interestingly, in the high-sugar diet group, deterioration of the long-term and short-term memory could be observed compared to mice on a normal diet [118].

## 5. Role of Supplementation on Cognitive Functions

It is widely recognized that cognitive health may be supported by maintaining a healthy lifestyle, comprising a balanced diet, regular physical activity, and mental engagement, thus prolonging life expectancy and delaying the onset of dementia. Additionally, the role of dietary supplementation has become increasingly significant. In recent years, studies have emphasized the potential role of nutritional supplements in supporting and enhancing cognitive function [119,120].

Research has shown the beneficial effects of folic acid, vitamin B12, and vitamin B6 in maintaining cognitive function [121]. Ma et al. found that 24-month folic acid (FA) supplementation improves cognitive function in mild cognitive impairment (MCI) [122]. The role of FA and B12 supplementation in elderly healthy people or people with any dementia or cognitive impairment was studied. FA and vitamin B12 supplementation showed a positive therapeutic effect in improving cognitive functions. When considering the combination of FA and vitamin B12, it was noted that it was significantly superior to either folic acid or vitamin B12 alone [123,124,125]. Elevated serum homocysteine levels have been associated with the development of Alzheimer’s dementia. The study by Sun et al. determined that supplementation with vitamins B6, B12, and FA decreased the homocysteine concentrations in patients with mild to moderate Alzheimer’s disease [126].

Further, these studies confirmed that supplementation with FA and docosahexaenoic acid (DHA), either alone or combined, significantly improves cognitive function and reduces plasma inflammatory cytokines in individuals with MCI. However, the combined use of FA and DHA is more effective than each nutrient taken separately [127]. Zhang et al. conducted a study that suggests that DHA supplementation (2 g/day) for 12 months in individuals with MCI can significantly enhance cognitive function and slow the progression of hippocampal atrophy [128,129].

Additionally, it has been shown that in healthy people, omega-3 fatty acids support cognitive performance and brain health [130,131,132]. Fatty acids, in particular, eicosapentaenoic acid (EPA) and DHA, are essential components of brain cell membranes and play a role in several neurophysiological functions [133]. DHA, the most prevalent omega-3 fatty acid in the brain, is well recognized for its contribution to the enhancement of neuronal signaling and synaptic plasticity, two essential components of cognitive functions like learning and memory [134]. Moreover, EPA supports cognitive health through its anti-inflammatory abilities, which helps to lessen neuroinflammation, which is a factor in both cognitive decline and the onset of neurodegenerative diseases [135]. Furthermore, increasing dietary consumption of omega-3 fatty acids and carotenoids may help lower the risk of dementia and cognitive deterioration in later life [136,137]. Nonetheless, these studies have shown no statistically significant effect on cognitive function [138,139,140,141]. Further research is needed to establish the complex interplay between omega-3 fatty acids supplementation and MCI pathophysiology, and therefore, to develop novel effective therapeutic interventions.

Adequate levels of vitamin D may support cognitive health, potentially improving memory and overall cognitive performance, particularly in older adults [142,143]. Supplementation is especially important for the health of the cerebrovascular system, as vitamin D lowers the risk of cerebrovascular diseases, which can affect cognitive function and maintain blood flow to the brain [144]. It has promise as a critical nutrient in preserving brain health through its roles in neuroprotection, neurotransmission, and brain plasticity [145,146]. Yang et al. noted that vitamin D in adults with MCI improves cognitive function through reducing oxidative stress regulated by increased telomere length [147]. Yet while these findings are encouraging, more research is required to definitively prove the link between vitamin D supplementations.

Maintaining cognitive health requires minerals like copper, zinc, iron, magnesium, selenium, and zinc, especially in the elderly population. Supplementing with them can help with a range of brain functions, including cognitive function, and lower the risk of neurodegenerative disorders [148].

On the contrary, the meta-analysis by McCleery et al. included 28 studies with over 83,000 participants and found limited evidence that vitamin or mineral supplements significantly improve cognitive function or reduce the dementia risk. B vitamins, antioxidant vitamins, vitamin D, calcium, zinc, and copper showed little to no effect on cognitive function in healthy older adults. The studies generally had low- to moderate-certainty evidence, and adverse events were rarely reported. While there was some evidence of benefit from long-term antioxidant use, overall, the supplements did not have a significant impact on cognition or dementia prevention [149].

Nutritional supplements can play a supportive role in enhancing cognitive function, particularly in cases of dietary insufficiencies or increased demands on brain health. B vitamins, omega-3 fatty acids, B vitamins, and D vitamins all show promise in improving aspects of cognition such as memory, attention, and mood. Further investigation is warranted to advance our knowledge and establish novel treatments and preventive strategies. Thus, the benefits of supplements should be considered as part of a broader approach to cognitive health, including a balanced diet, regular physical activity, and mental exercises.

## 6. Potential Microbiota-Targeted Therapies for Cognitive Impairment Disorders

Research has demonstrated that individuals with PD and AD often exhibit altered gut microbiota profiles, characterized by a decrease in beneficial bacteria and an increase in pro-inflammatory species. This state may contribute to the neuroinflammatory processes and neurodegeneration observed in these conditions [150,151]. Targeting the gut microbiota to restore the balance may thus have significant therapeutic implications.

Prebiotics are food components that are selectively utilized by host bacteria, providing health benefits. According to Liu et al.’s findings, mannan oligosaccharide (MOS) improves behavioral and cognitive impairments, possibly via altering the gut microbiome and increasing SCFA production, proposing MOS as a potential microbiota-targeted therapy for neurodegenerative illnesses such as Alzheimer’s. MOS significantly reduces Aβ accumulation in the cortex, hippocampus, and amygdala, while also balancing brain redox status and suppressing neuroinflammatory responses. Additionally, MOS alleviates HPA axis dysfunction by lowering the corticosterone (CORT) and corticotropin-releasing hormone (CRH) levels while increasing norepinephrine (NE) expression [152].

Probiotics are live microorganisms, which, when consumed in adequate amounts, provide health advantages to the host. Interestingly, probiotics have been shown in both PD and AD models to reduce neuroinflammation, improve gut barrier integrity, and increase synthesis of neuroprotective metabolites such SCFAs [150,151]. According to Castelli et al., supplementing with a probiotic cocktail that included Lactobacillus acidophilus, Bifidobacterium animalis lactis, and L. rhamnosus GG increased butyrate synthesis, which in turn decreased the loss of nigral dopaminergic neurons [153]. Nimgampalle et al. examined the effects of Lactobacillus plantarum MTCC 1325 on D-galactose-induced AD in mice. The findings revealed a decrease in neurofibrillary tangles (NFTs) and amyloid plaques, along with increased acetylcholine (ACh) levels in the hippocampus and cerebral cortex. Additionally, the memory and spatial learning were improved in the treated mice. These effects are likely due to the production of ACh by Lactobacillus plantarum MTCC 1325, a neurotransmitter with potent antioxidant properties that may stabilize the amyloid-beta structure and reduce Aβ aggregation [154].

FMT has emerged as a promising therapeutic intervention for a range of gastrointestinal disorders, primarily focusing on conditions like Clostridioides difficile infection [155]; however, its potential associations with cognitive impairment are drawing increasing attention in the field of neurogastroenterology. Recent studies suggest that the gut–brain axis, a bidirectional communication system between the gastrointestinal tract and the central nervous system, plays a crucial role in cognitive functions and mental health [156,157]. Alterations in the gut microbiota composition have been linked to various neurological conditions, including Alzheimer’s disease [158]. Emerging research indicates that restoring microbial diversity through FMT may influence neuroprotective pathways, modulate inflammatory responses, and impact neurotransmitter levels, including those of serotonin and dopamine, which are vital for cognitive health [159,160]. Animal studies have shown that FMT can improve cognitive function in models of neurodegeneration, suggesting that the gut microbiota may directly influence neurodevelopment and neuroplasticity. While human studies remain limited, preliminary findings indicate potential improvements in cognitive performance and mood in individuals undergoing FMT for conditions associated with dysbiosis [161,162]. As the understanding of the complex interplay between the gut microbiota and brain health evolves, these findings may provide new therapeutic strategies for managing cognitive impairment, although further research is crucial.

## 7. Conclusions

In conclusion, the underlying processes through which dietary intake influences cognitive function are complex and multifaceted, involving the modulation of neuroinflammation, oxidative stress, synaptic plasticity, and, increasingly recognized, the gut–brain axis [81]. Nutrient-rich diets, particularly those high in omega-3 fatty acids, antioxidants, and vitamins, are linked to improved cognitive function, delayed decline, and reduced risk of neurodegenerative diseases [163,164]. In contrast, diets rich in saturated fats, sugars, and processed foods contribute to cognitive impairment and dementia [165,166]. The gut–brain axis plays a critical role, as a healthy gut microbiome supports brain health, while poor nutrition disrupts this balance. Holistic dietary patterns like the Mediterranean and DASH diets, rich in fruits, vegetables, whole grains, and healthy fats, benefit both brain and gut health, enhancing overall cognitive function [167].

However, while the correlation between diet and cognitive function is compelling, establishing causality remains challenging due to the inherent complexity of diet-related research, the influence of confounding variables, and the long-term nature of cognitive outcomes. Future research should aim to further elucidate these mechanisms, particularly the role of the gut–brain axis, employ more rigorous methodologies, and explore the potential of personalized nutrition interventions to optimize cognitive function.
